# Reply to comment on “A jumbo cyanophage encodes the most comprehensive ribosomal protein set in the known virosphere” by LinXing Chen

**DOI:** 10.1093/ismejo/wrag242

**Published:** 2026-09-30

**Authors:** Isaac Meza-Padilla, Sarit Avrani, Kirsten M Müller, Jozef I Nissimov

**Affiliations:** Department of Biology, University of Waterloo, 200 University Avenue West, Waterloo, Ontario N2L 3G1, Canada; Department of Evolutionary and Environmental Biology and Institute of Evolution, University of Haifa, Haifa 3498838, Israel; Department of Biology, University of Waterloo, 200 University Avenue West, Waterloo, Ontario N2L 3G1, Canada; Department of Biology, University of Waterloo, 200 University Avenue West, Waterloo, Ontario N2L 3G1, Canada

**Keywords:** ribosome, cyanophage, jumbo phage, prophage, *Vampirovibrio*, *Microcystis*, horizontal gene transfer

In our recent article titled “A jumbo cyanophage encodes the most comprehensive ribosomal protein set in the known virosphere” [1], we reported the identification of a large set of ribosomal proteins (RPs) in the jumbo cyanophage PhiMa05 and similar prophages in *Vampirovibrio* genomes. We also proposed an evolutionary scenario to explain the ultimate causes and consequences of RP acquisition by PhiMa05. Subsequently, Chen [2] raised questions on the validity of our findings, focusing on whether PhiMa05 is indeed a complete cyanophage genome, therefore putting in doubt our proposed evolutionary hypothesis.

We agree with Chen’s broader concern that overinterpretation of cellular-looking sequences as viral is a real risk in the current era of large-scale, automated metagenomic annotation. Where we differ is in the conclusion that this particular genome, PhiMa05, is best explained as a fragment of a bacterial genome or non-viral genetic element, instead of a complete cyanophage genome. First and foremost, the PhiMa05 genome is not a metagenomically-derived contig computationally categorized as viral, but the complete genome of an isolated virus, sequenced at 92× coverage, and cultivated in a laboratory [3]. Considered together, the evidence already published in our study, the isolation methodology used to generate the sequenced material by Naknaen *et al*. [3], and the current well-documented limitations of automated viral classifiers and structural databases for jumbo phages and divergent viral genomes [4–7] make the cyanophage genome interpretation of PhiMa05 the most likely explanation.

## Independent whole-genome proteomic, portal protein, and major capsid protein phylogenies place PhiMa05 among cyanophages

Our assignment of PhiMa05 as a cyanophage rests on three independent phylogenies: whole-genome proteomic [8], major capsid protein [3], and portal protein trees [1]. These are precisely the hallmark structural genes and genomic content lines of evidence that Chen correctly argues should be taken into consideration. As Chen appropriately points out, viral prediction tools can miss highly divergent and “unusual” viruses. A non-virus call from geNomad or VirSorter2 does not outweigh three concordant and independent marker genes and whole-genome proteomic phylogenies [1, 3, 8] that consistently and specifically place PhiMa05 within existing cyanophages, including a recognized viral family, rather than simply flagging generic phage-like domains.

In particular, our maximum likelihood phylogenetic reconstruction strongly (i.e. support value of 100 out of 100 bootstrap replicates) supports not only PhiMa05 but also the PhiMa05-like prophages in *Vampirovibrio* genomes to be closely related to saffermanviruses [1]. The *Saffermanviridae* is a lineage of temperate freshwater cyanophages comprised of both free viruses and prophages [9] that is formally recognized by the International Committee on Taxonomy of Viruses as a viral family in the class *Caudoviricetes* [10]. Chen argues that the genomic content of PhiMa05 is one of the main lines of evidence supporting its classification as a bacterial genome fragment or non-viral genetic element. Therefore, we would like to emphasize the whole-genome proteomic tree, conducted by an independent research group, which again places PhiMa05 among freshwater cyanophages [8]. In other words, the PhiMa05 genome clusters with other freshwater cyanophage genomes when considering all its protein-coding genes.

## Independent identification of the same genomic region in different host genomes argues strongly against a one-off contamination event

Critical evidence against the contamination/co-purified fragment hypothesis from Chen is one we already reported: a PhiMa05-like prophage carrying the same conserved RP genes and viral structural proteins occurs, with clear synteny, in at least two independently isolated and sequenced genomes of *Vampirovibrio chlorellavorus* (Vc_AZ_1 and Vc_AZ_2) [1]. These *V. chlorellavorus* strains were isolated in different years (March 2016 and May 2017) from experimental algal cultivation ponds in Arizona, USA—a study, place, and dates of isolation entirely unrelated to the Thailand wastewater isolation of PhiMa05 in 2020 [3, 11]. In Vc_AZ_1, the PhiMa05-like prophage is flanked by host genomic sequence on both sides, and in Vc_AZ_2 it is flanked by host sequence on one side with the contig ending inside the prophage region on the other. A contamination or misassembly artifact cannot explain why the same gene cluster, in the same order, recurs as an integrated element inside two independently assembled bacterial chromosomes from a different laboratory, host species, and continent. The additional observation that bL33 is specifically pseudogenized within the Vc_AZ_2 prophage, while being present elsewhere in that same genome, is itself a signature of an evolving, integrated mobile genetic element under selection, not a static co-purified fragment.

We note that this is a stronger form of corroborating evidence than the single distantly homologous locus Chen offers in support of the alternative interpretation: a portal protein- and major capsid protein-containing scaffold from one metagenome-assembled *Candidatus Melainabacteria* genome recovered from a landfill sample. Metagenome-assembled scaffolds of this kind carry their own, well-known risks of chimerism and miss-binning, and a single distant locus from an unrelated dataset is not, in our view, sufficient to override the coherent, repeated, multi-genome pattern we report for PhiMa05 and related prophages in *Vampirovibrio*. We reiterate that PhiMa05, Vc_AZ_1, and Vc_AZ_2 are not metagenome-assembled genomes, but microbes that were independently isolated and cultivated before sequencing. A distant, structurally simpler trace in a metagenome-assembled bacterial scaffold by no means demonstrates that PhiMa05 is non-viral.

## Automated virus identification tools face well-documented challenges with divergent and poorly referenced viral genomes

Of the 256 open reading frames (ORFs) in the PhiMa05 genome, many are hypothetical proteins [1–3]. For example, in the original isolation study only 54 ORFs could be functionally annotated [3]. This is a hallmark of jumbo phages, whose genomes are well-known to be divergent and poorly represented in genomic databases [4]. It is exactly the kind of genome that poses challenges for virus identification tools, including geNomad and VirSorter2, as performance tends to drop among sequences that are divergent from the training and reference data [5, 6]. Notably, Chen’s own recent work independently raises the same point: the study of jumbo phages “has been hindered by challenges in genome identification and the lack of a comprehensive reference database” [7]. We would suggest that a negative virus identification call from automated computational tools and an apparent incomplete ColabFold-based structural picture for PhiMa05 are more consistent with these known detection gaps for divergent and poorly referenced viral genomes than with a non-viral origin.

## A note on the structural proteins of PhiMa05

Chen states that the only structural proteins detected in the PhiMa05 genome are a major capsid protein and a portal protein and argues that this supports their claim that PhiMa05 is not a virus. However, this is not the case. A major capsid protein and a portal protein are not the only structural protein-coding genes present in PhiMa05. The viral genome also encodes homologs of phage adaptor, packaged DNA stabilization, and tail fiber proteins [1, 3]. Given that jumbo phage proteins are highly divergent and often have little similarity to those present in databases [4], many proteins can easily remain undetected during computational annotation efforts. Yet, several viral structural proteins have indeed been identified in PhiMa05. Please also note that Fig. S1 in our article [1] does not display a “structural comparison,” as incorrectly stated by Chen, but a maximum likelihood phylogeny reconstructed from amino acid sequences of portal proteins.

## Cellular-like genes are a characteristic feature of jumbo phage genomes, not evidence against viral status

As accurately captured in a recent review, “Jumbo phages challenge long-standing notions of phage simplicity, redefining the boundaries of what a phage can be” [4]. Jumbo phages have repeatedly been found to carry an unusually broad “cellular-like” gene repertoire, including components of DNA replication, repair, transcription, nucleotide metabolism, and protein modification [12]. Furthermore, bacterial flagellar motor-associated and Fe-S cluster assembly genes have been reported previously in viruses and fall well within the diversity of auxiliary metabolic functions that viral genomes can and have been found to encode [13, 14].

## On the purification and sequencing workflow, and the contamination hypothesis

We do not think that the co-purified DNA fragment hypothesis, proposed by Chen, is compatible with how PhiMa05 was isolated and sequenced. Naknaen *et al*. [3] purified PhiMa05 virions from lysates using both filtration and a conventional cesium chloride density step gradient, a physical isolation method that isolates intact virus particles by density and excludes free, non-encapsidated DNA that would not co-sediment at the same density. Furthermore, the cyanophage lysate was treated with DNase I and RNase A to remove any remaining non-encapsidated nucleic acid prior to phage DNA extraction and Illumina NovaSeq sequencing. Chen states that “Viromics workflows typically reduce contamination from cellular DNA through filtration, nuclease treatment, and density-gradient purification prior to sequencing.” That is exactly what was done in the case of PhiMa05. Critically, a “sharp single DNA band” was reported after DNA quality check via electrophoresis [3], pointing strongly against the possibility of any co-purified DNA fragments. This workflow, from repeated phage propagation to density-gradient purification and nuclease treatment, is specifically used to exclude the kind of contamination that Chen proposes as an alternative explanation; it is a materially different starting point from a mixed-community metagenomic co-assembly, where such contamination is a genuine and well-known risk [15].

The original isolation study did not deposit raw Illumina reads, and this unfortunately limits fully independent read-level reanalysis. However, it is important to remember that this is a limitation shared by many viral genome depositions. Also note that the PhiMa05 genome was deposited in NCBI in 2021 and then verified in 2023 (see the Revision History section of the GenBank accession number for PhiMa05, MW495066.1). If possible, we hope to pursue read-level reanalysis and/or long-read resequencing by obtaining the phage, in collaboration with the group that isolated it, as a valuable next step for the field. As continuation of this work, we also hope to obtain the two *Vampirovibrio* lysogens to experimentally investigate them further. Collecting and analyzing cell-associated and free viral metagenomic samples from the places of isolation of PhiMa05 and the *Vampirovibrio* and *Microcystis* hosts also holds great potential for future investigations.

## Conclusion

We remain grateful for the opportunity this exchange gives the field to sharpen the standards used to validate unusual genomes of cultivated viruses, and we agree that no single line of evidence should be treated as sufficient on its own. However, taken together, (i) the three independent, hallmark gene, and whole-genome proteomic phylogenies placing PhiMa05 within cyanophages, including an established temperate freshwater cyanophage family; (ii) the independent syntenic replication of the same RP and viral structural proteins-containing genomic region, integrated into host DNA, across two *Vampriovibrio* genomes from a separate study and continent; (iii) the well-documented detection gaps of virus identification tools and structural databases for divergent jumbo phages; (iv) the presence of various viral structural protein-coding genes in PhiMa05; (v) the established precedent that broad “cellular-like” gene content is a characteristic feature of jumbo phage genomes rather than a marker of contamination; and (vi) a virion purification and nuclease-treatment workflow specifically designed to exclude contaminating DNA, support that PhiMa05 is indeed a cyanophage genome. We share Chen’s view that additional analyses, including raw-read reanalysis and long-read resequencing, would further strengthen this conclusion, and we welcome continued scrutiny of PhiMa05 and of similar unusual genomes of cultivated viruses as the field’s tools and reference databases improve.

## Data Availability

No data was generated during this study.
